# Supplementary material for: Patterns of Intron Gain and Loss in Fungi
Source: PLoS Biol. 2004 Nov 30;2(12):e422. doi: 10.1371/journal.pbio.0020422 (PMC532390; doi:10.1371/journal.pbio.0020422)
Supplement: Table S1 — Also available at http://genes.mit.edu/NielsenEtAl/. (4.3 MB ZIP). [file pbio.0020422.st001.zip › NielsenEtAl/html/752.html]

AN2260.1.NCU01142.1.MG07542.1.FG09005.1


```
 CLUSTAL W (1.82) Multiple Sequence Alignments - Introns Inserted


Sequence 1: NCU01142.1	117 aa
Sequence 2: FG09005.1	136 aa
Sequence 3: MG07542.1	136 aa
Sequence 4: AN2260.1	146 aa
Alignment Length: 150 aa
Number Identitical Residues: 49 aa
Alignment Score (without introns) 2889


MG07542.1 	MSTLSRTFGYLRRIGIK0EYWHQLN0------~----YIG1DTKAGTLIGTDRFGNKFYE
NCU01142.1	-----------------~-----MN0------~----YIG1DTKAGTLVGVDKFGNKFFE
FG09005.1 	MSTVGRTLTNLRKVGIK0DYFKQML0------~----YIG1DTKAGVLIGTDRAGNKYFE
AN2260.1  	MSTILRTLRNLRRIGFK~VGYTATE~TAAPIG1IWPPNAG1DTKAGTLIGTDRYGNKYYE
          	 ::   :       . .         :::. .   ..  * *****.*:*.*: ***::*

MG07542.1 	NMAEELPL1RTRWVDYKKHDYDA2AHIEPHW~HAWLSYMVDTPAPQDALTKIN--RPWAP
NCU01142.1	NQ-EELPL~RTRWVDFAKHDYDP~SHIEPLW~HAWISYAIDTVPTQDPIATQAPKRPWAP
FG09005.1 	NN-EELPL~RTRWVEYAVHDYDA~AHIEPGW~HAWMSYSVDKPPTQDPLLNAG-TRAFEP
AN2260.1  	NMEEELPL1RTRWVDYKQKEYDP~SQIEPGW2HAWISYMVDIPPTNDKIMQLG-VRPWEL
          	*  ***** *****::  ::**. ::*** * ***:** :*  ..:* :      *.:  

MG07542.1 	KEHVGNRTATRGAYKPYST2--TVPKFKAWEPRAAARS
NCU01142.1	EVHHPNYTTLPGAFKTFNT2SSVKPKIQSWEPVAAPRH
FG09005.1 	SRAIPNFTQTKGAFKTYNT2--AKPKMATWQPVAAPRE
AN2260.1  	REHRPTLTLSRAAFKTYST2--TKPKYTAWNPVAAPR-
          	     . *   .*:*.:.*   . **  :*:* **.*
```
